# Hymeneal

**Published:** 1856-01

**Authors:** 


*Hymeneal.*—Married, at Richmond, Virginia, recently, Mr. "Stethoscope to Miss Virginia Medical Journal." These parties were from the "first families" of the Old Dominion, and as they are now of "one flesh," we shall expect regular and interesting issues.

